# Supplementary material for: Complex Age- and Cancer-Related Changes in Human Blood Transcriptome—Implications for Pan-Cancer Diagnostics
Source: Front Genet. 2021 Oct 15;12:746879. doi: 10.3389/fgene.2021.746879 (PMC8554330; doi:10.3389/fgene.2021.746879)
Supplement: Supplementary file 3 [file DataSheet1.pdf]

Supplementary Figures

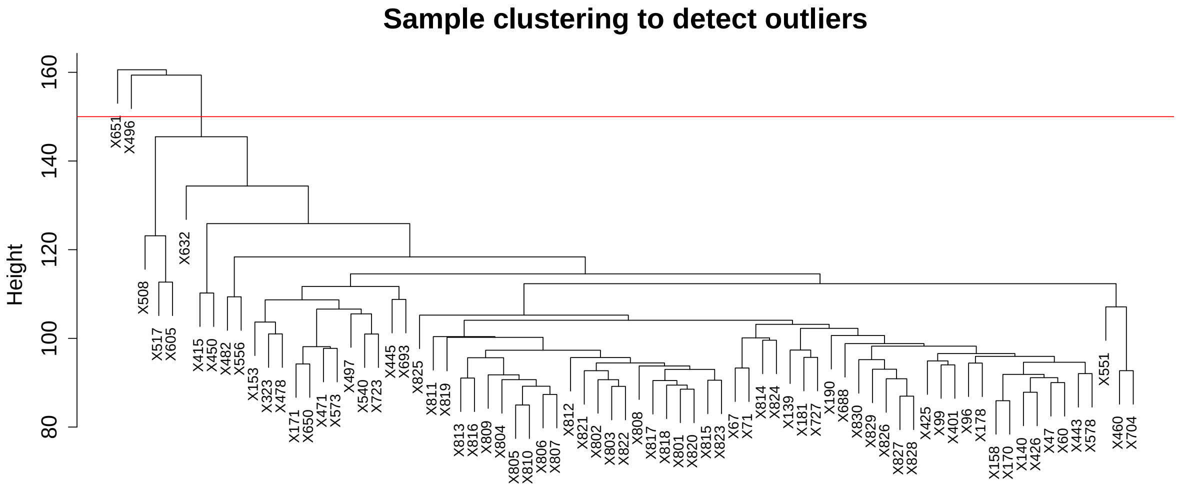

Supplementary Figure 1 Clustering of the samples to detect outliers in the gene co-expression network analysis. The red line indicates the cutoff for outliers.

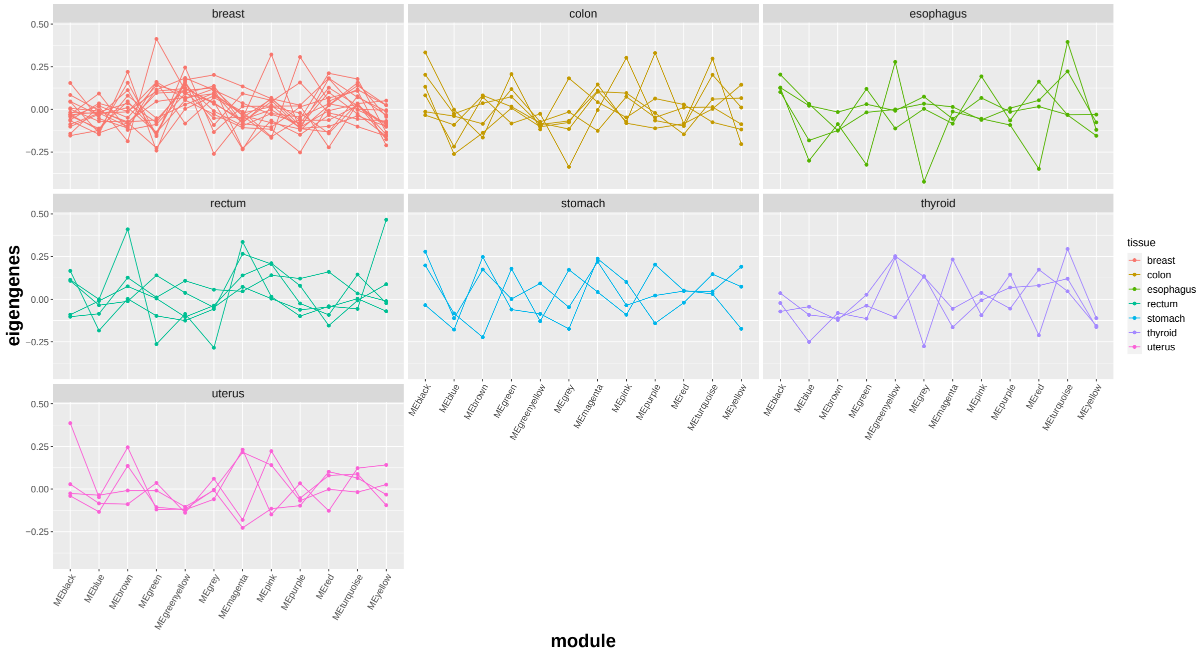

Supplementary Figure 2 No correlation was observed between the co-expression modules and the tissue of cancer origin. The y-axis shows the value of eigengenes of each module representing the expression levels. Each line represents one sample.

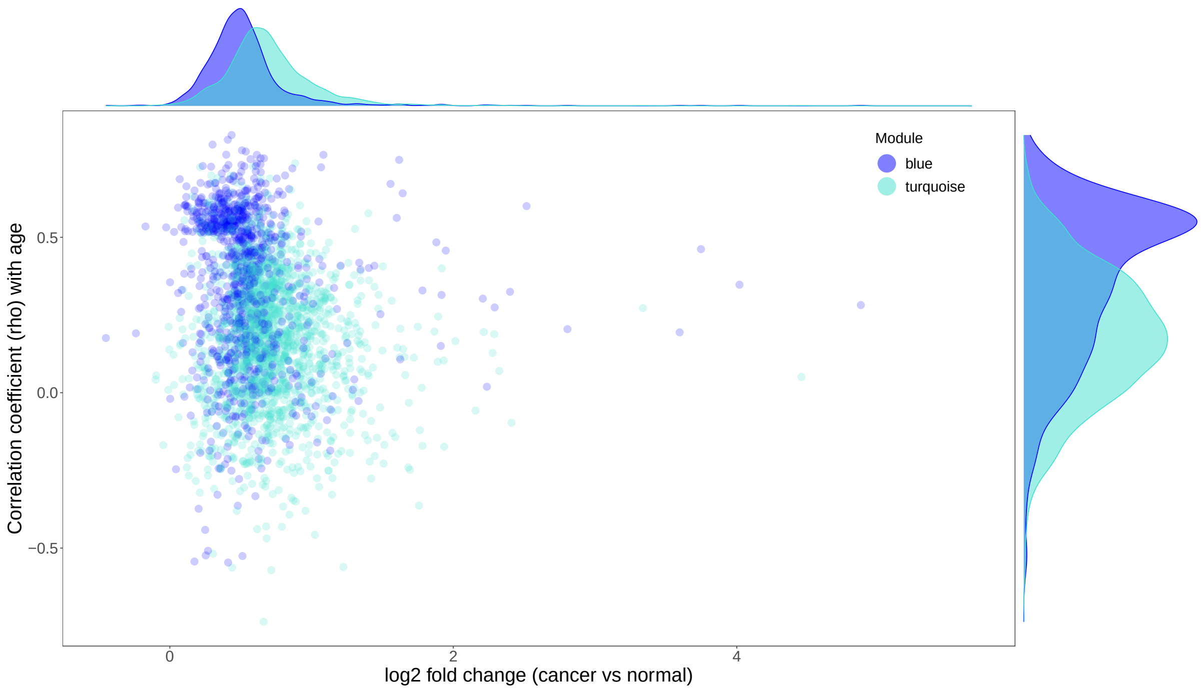

Supplementary Figure 3 The magnitude of age- and cancer-related changes were different between the up- (module turquoise) and down-regulated (module blue) genes. This figure is the same as Figure 7, but the Spearman correlation coefficients and log2 fold changes of down-regulated genes (module blue) were inverse (multiplied by -1).

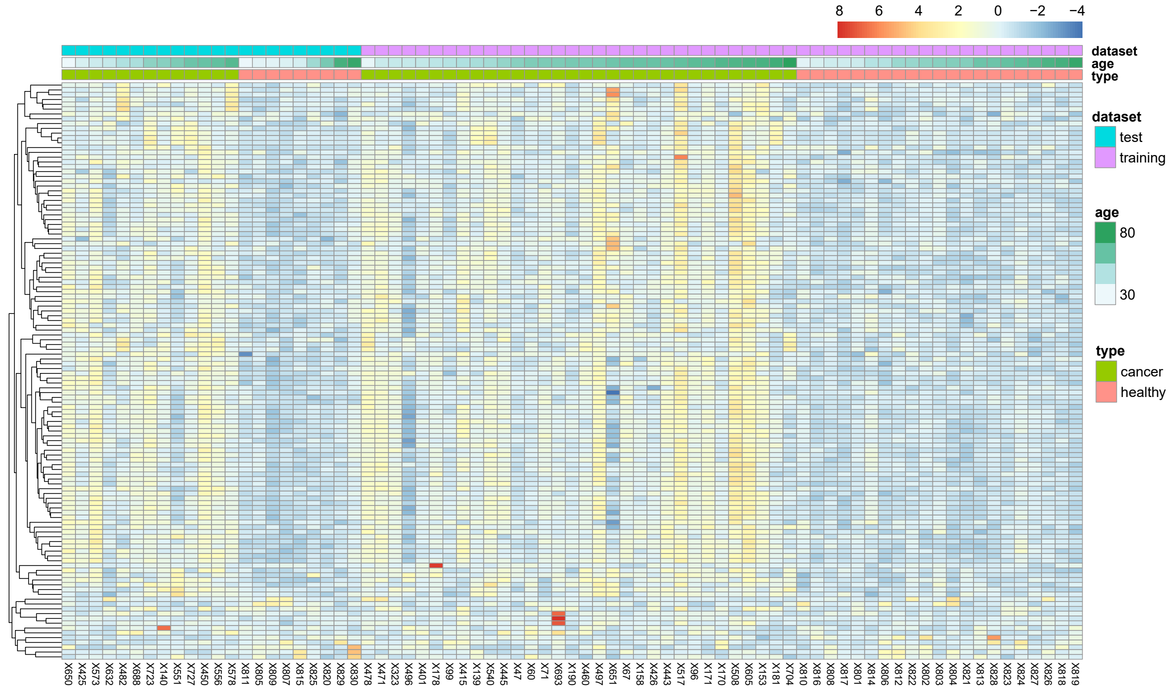

Supplementary Figure 4 Heatmap of the expression levels of the 120 vlinRNAs which are differentially expressed between then cancer and normal samples of the training dataset and used to build the vlinc-classifier. The expression levels were Z-scores of variance stabilizing transformed read counts.
